# Time Pressure Modulates Electrophysiological Correlates of Early Visual Processing

**DOI:** 10.1371/journal.pone.0001675

**Published:** 2008-02-27

**Authors:** Ingo Fründ, Niko A. Busch, Jeanette Schadow, Thomas Gruber, Ursula Körner, Christoph S. Herrmann

**Affiliations:** 1 Institute of Psychology, Department of Biological Psychology, Otto-von-Guericke University, Magdeburg, Germany; 2 Bernstein Group for Computational Neuroscience, Magdeburg, Germany; 3 Leibniz Institute for Neurobiology, Magdeburg, Germany; 4 Centre de Recherche Cerveau et Cognition, Centre National de la Recherche Scientifique (CNRS), Université Paul Sabatier, Toulouse, France; 5 Institute of Psychology I, University of Leipzig, Leipzig, Germany; 6 Honda Research Institute, Offenbach, Germany; 7 Center for Behavioral Brain Sciences, Magdeburg, Germany; Baylor College of Medicine, United States of America

## Abstract

**Background:**

Reactions to sensory events sometimes require quick responses whereas at other times they require a high degree of accuracy–usually resulting in slower responses. It is important to understand whether visual processing under different response speed requirements employs different neural mechanisms.

**Methodology/Principal Findings:**

We asked participants to classify visual patterns with different levels of detail as real-world or non-sense objects. In one condition, participants were to respond immediately, whereas in the other they responded after a delay of 1 second. As expected, participants performed more accurately in delayed response trials. This effect was pronounced for stimuli with a high level of detail. These behavioral effects were accompanied by modulations of stimulus related EEG gamma oscillations which are an electrophysiological correlate of early visual processing. In trials requiring speeded responses, early stimulus-locked oscillations discriminated real-world and non-sense objects irrespective of the level of detail. For stimuli with a higher level of detail, oscillatory power in a later time window discriminated real-world and non-sense objects irrespective of response speed requirements.

**Conclusions/Significance:**

Thus, it seems plausible to assume that different response speed requirements trigger different dynamics of processing.

## Introduction

Actions are continuously adjusted to sensory input. This requires fast processing of sensory stimuli in order to make them available for motor reactions. At the same time, many situations require more detailed analyses for refinement of perceptual outcome and adaptation of future behavior [1]. Are these functions governed by the same neural system or do we employ different systems for these tasks?

Processing speed could be achieved by rapid feedforward categorization of incoming stimuli [2], [3]. More detailed analyses seem to rely on interareal feedback refining these initial categories [2], [4]. Recently, Herrmann et al. [5] linked these two modes of processing to electroencephalographic (EEG) gamma band oscillations. Rapid categorization seems accompanied by early stimulus-locked, so-called evoked gamma band responses (eGBRs, latency ∼100 ms, gamma band: 30–90 Hz). Later refinement of these categories appears linked to late induced gamma band responses (iGBRs, latency ∼300 ms). Response characteristics of evoked and induced GBRs differ. Evoked GBRs highly depend on physical stimulus salience [6], [7] and are modulated by attention [8], [9]. They increase after matches between sensory input and experience based object templates [10], [11]. Such matches were assumed to result in (i) more efficient information transfer to later stages of processing, and (ii) enhanced feedback signals from the locus of the match. These local feedback signals could reverberate between low level, feature sensitive visual areas at gamma frequencies [5]. In contrast, iGBRs seem related to semantic stimulus content [12], [13], [14]. If a semantic representation for a particular stimulus class emerges during multiple presentations, increasingly strong iGBRs were observed [13]. Evoked and induced GBRs also display different dynamics. While eGBRs are mainly explained by increased stimulus-locking [8], [7], [15], iGBRs occur as amplitude increases with varying latency after the stimulus [16], [17]. Since timing as manifested in stimulus-locking is the first available information about a stimulus [18], a fast processing mechanism is likely to employ timing information to differentiate stimuli [19]. These data suggest that eGBRs might reflect fast processing of sensory information [20], whereas iGBRs might reflect more elaborated processing and integration of sensory information with previous knowledge [2], which we tested in this study.

**Figure 1:**
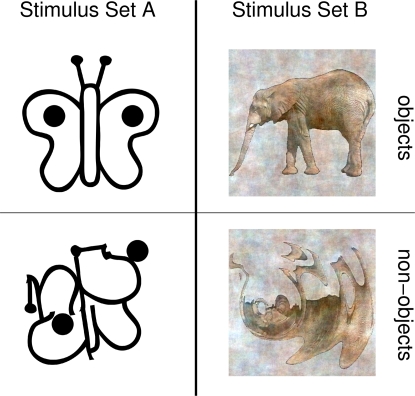
Example stimuli employed in the current experiment. Left: an example of a real-world and a non-sense object stimulus from stimulus Set A. Right: an example of a real-world and a non-sense object stimulus from stimulus Set B.

We compared speeded and delayed responses and expected only the former to modulate eGBRs, since speeded responses can only be based on fast stimulus discriminations. We compared processing of schematic and detailed visual stimuli like those in Figure 1, and expected that only the latter modulate iGBRs, since the refinement of an initial rough categorization would only be possible for more detailed stimuli. If eGBRs were associated with rapid stimulus discrimination, eGBR modulations should be particularly salient in speeded response tasks that can only be based on rapid stimulus discriminations. We expected that further refinement of the initial rough categorization would only be possible for more detailed stimuli containing more potentially diagnostic features. If such refinement processes were related to iGBRs, iGBR modulations should be more pronounced with more detailed stimuli.

## Results

### Behavioral data

**Figure 2:**
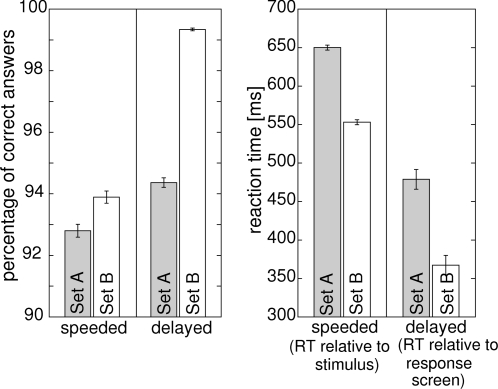
Behavioral data in the current experiment. Left display: percentages of correct responses. Right display: reaction times. Results from schematic line drawings (Set A) are represented by gray bars. Results from colored, more detailed images (Set B) are represented by white bars. Note, that the accuracy benefit from the additional information contained in stimuli from Set B is much more pronounced for delayed responses. Note also, that reaction times in the speeded task refer to stimulus onset, while in the delayed task reaction times refer to the onset of the response screen which appeared 1000 ms after stimulus onset.

Mean reaction times were generally faster for the more detailed stimuli from Set B than for the line drawings from stimulus Set A (main effect of LEVEL OF DETAIL: F_1,16_ = 21.03, p = 3.05*10^−4^, repeated measurements ANOVA with factors LEVEL OF DETAIL, TIME PRESSURE, and SEMANTIC CONTENT). In addition, mean reaction times to the response screen (one second after stimulus onset) in the delayed condition were on average 178 ms shorter than to the stimulus in the speeded response condition (main effect of TIME PRESSURE: F_1,16_ = 322.87, p = 4.96*10^−12^). In the speeded response task, participants made significantly more errors compared to the delayed response task (main effect of TIME PRESSURE: F_1,16_ = 34.63, p = 2.30*10^−5^, ANOVA details as for reaction times). Participants also made more errors in response to line drawings from stimulus Set A as compared to the more detailed stimuli from stimulus Set B (main effect of LEVEL OF DETAIL: F_1,16_ = 23.06, p = 1.95*10^−4^). This was particularly true in the delayed response task (LEVEL OF DETAIL×TIME PRESSURE interaction: F_1,16_ = 12.14, p = 0.003). See Figure 2 for a summary of the behavioral results.

### Event related potentials

**Figure 3:**
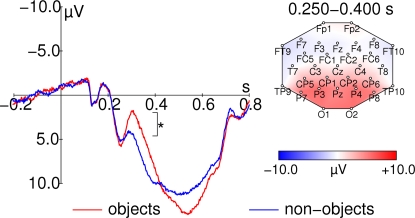
Event related potentials. Left: ERP-waveform at Pz for object (red) and non-sense object (blue) stimuli. Stimulus onset is at 0 ms. Time courses have been normalized by subtracting the average potential from the last 200 ms before stimulus onset. Right: topographic map of averaged activity from all conditions between 250 and 400 ms after stimulus onset. Data from both stimulus sets have been averaged in this figure. Note, that the clear late negative deflection is observed for object stimuli only. The asterisk indicates a significant difference that is described in the text.

A late modulation of the event related potential (ERP) was observed between 250 and 400 ms (see Figure 3 left). A prominent effect in this time window was a strong modulation of the average amplitude in the time range 250 to 400 ms when the stimuli were real-world objects (main effect SEMANTIC CONTENT: F_1,16_ = 151.27, p = 1.44*10^−9^, ANOVA details as for behavioral data).

### Early gamma band response

**Figure 4:**
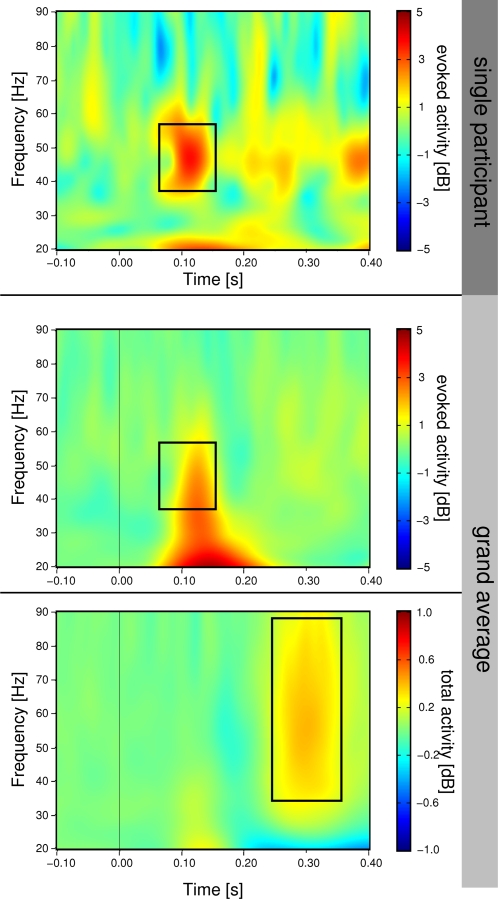
Time frequency representations of oscillatory activity. Top: evoked activity from a single representative participant. Middle: evoked activity averaged across all participants. Bottom: total activity averaged across all participants. Note, how averaging of evoked activity smeares the relatively focal activity of single participants (black boxes in top and middle display). These data have been obtained by averaging time frequency planes from the posterior ROI. Stimulus onset is at 0 ms. Amplitudes are in dB relative to a baseline 200 up to 100 ms before stimulus onset.

Figure 4 displays time frequency representations of evoked oscillatory activity in a single participant (top) and averages across all participants (middle). A clear eGBR can be observed for the single participant, which is smeared in the averaged activity due to considerable variance in the response frequencies between subjects (compare activity in the boxes in Figure 4). In order to perform statistical analyses of this activity, time courses at the peak frequency of the response were selected for further analysis [7]. Statistical significance was accessed by means of an ANOVA for repeated measurements with the factors LEVEL OF DETAIL (Stimulus Set A vs. Stimulus Set B), TIME PRESSURE (speeded vs. delayed), SEMANTIC CONTENT (real-world vs. non-sense objects), and region of interest (ROI, posterior vs. central).

**Figure 5:**
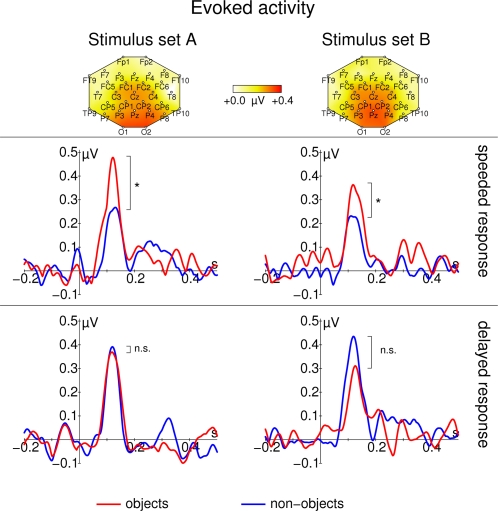
Early gamma band response for real-world (red) and non-sense objects (blue). Data from stimulus Set A (schematic black and white line drawings) are shown on the left; data from stimulus Set B (more detailed, colored images) are shown on the right. The top row of each panel shows topographic maps of the averaged activity from all conditions. Response frequencies and maps were determined from the time range 60–140 ms respectively. In the second row, responses for the speeded response task are shown. In the third row, responses for the delayed response task are shown. All time course data are taken from Pz. Note that eGBRs to real-world object stimuli are enhanced only if participants need to perform a speeded response. Stimulus onset is at 0 ms. Responses are normalized by subtracting the average activity between 200 and 100 ms before stimulus onset from the entire time course.

**Figure 6:**
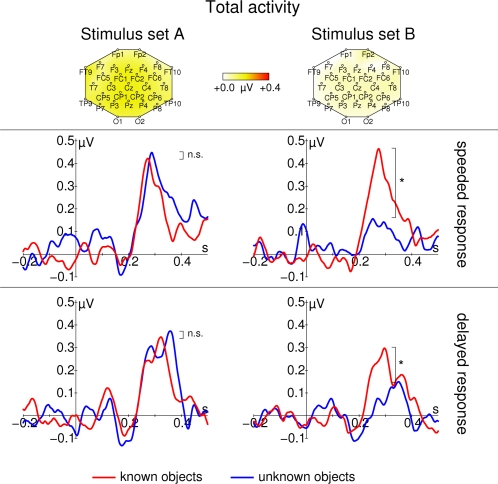
Late total gamma band response for real-world (red) and non-sense object (blue). Data from stimulus Set A (schematic black and white line drawings) are shown on the left; data from stimulus Set B (more detailed, colored images) are shown on the right. The top row of each panel shows topographic maps of the averaged activity from all conditions. Response frequencies and maps were determined from the time range 200–400 ms respectively. In the second row, responses for the speeded response task are shown. In the third row, responses for the delayed response task are shown. All time course data are taken from Pz. Total gamma band activity (f) responds in a highly stimulus specific way: Although strongest responses are observed for stimulus Set A, differences between real-world and non-sense object stimuli can only be found for the more detailed stimuli in stimulus Set B. Note, that there is no early response at all. Asterisks mark significant differences that are described in the text, n.s. denotes nonsignificant differences. Stimulus onset is at 0 ms. Responses are normalized by subtracting the average activity between 200 and 100 ms before stimulus onset from the entire time course.

**Figure 7:**
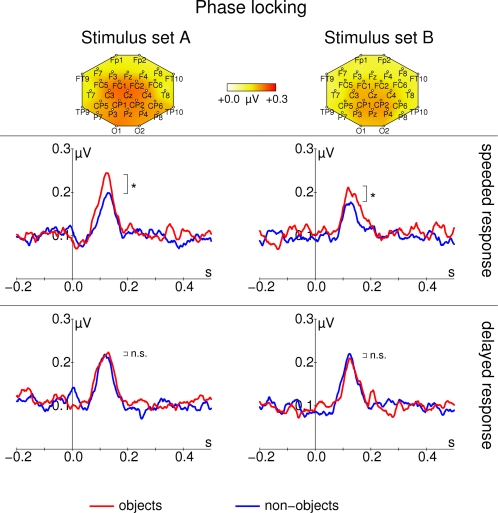
Phase locking (PL) of the early gamma band response to object (red) and non-sense object (blue) stimuli. Data from stimulus Set A (schematic black and white line drawings) are shown in the left column. Data from stimulus Set B (natural colored images) are shown in the right column. The top row shows topographic maps of the averaged phase locking from all conditions between 60 and 140 ms. In the second row, PL for the speeded response task is shown. In the third row, PL for the delayed response task is shown. All time course data are taken from Pz. Note, that PL after presentation of object stimuli is enhanced only if participants need to perform a speeded response. Response frequencies were determined from the time range 60 to 140 ms after stimulus onset. Asterisks mark significant differences that are described in the text, n.s. denotes nonsignificant differences. Stimulus onset is at 0 ms.

These evoked GBRs are depicted in Figure 5. An eGBR could be observed in all participants. The eGBR was not significantly different between posterior and central electrodes (F_1,16_ = 2.41, p = 0.140). The eGBR differed between real-world and non-sense object stimuli only if a speeded response was required (TIME PRESSURE×SEMANTIC CONTENT interaction: F_1,16_ = 5.00, p = 0.039, real-world vs. non-sense object effect for delayed responses: t_16_ = -0.77, p = 0.449, real-world vs. non-sense object effect for speeded responses: t_16_ = 2.29, p = 0.035). To differentiate between stimulus related changes in oscillatory amplitude and oscillatory phase, we calculated the average analytic amplitude across all trials (total oscillatory activity) and the phase locking factor [21]. The effect on eGBR was not accompanied by any effect of semantic content on total oscillatory activity (no significant effect in ANOVA, see Figure 6, although this displays results with frequencies adapted to the late response). Similar to evoked activity, phase locking of the early GBR to the stimulus differentiated real-world and non-sense object stimuli in the speeded response task (TIME PRESSURE×SEMANTIC CONTENT interaction: F_1,16_ = 4.96, p = 0.040, real-world vs. non-sense object effect for delayed responses: t_16_ = −0.41, p = 0.684, real-world vs. non-sense object effect for speeded responses: t_33_ = 2.59, p = 0.019, see Figure 7). Phase locking was also significantly enhanced at posterior electrodes (main effect of ROI: F_1,16_ = 32.28, p = 2.97*10^−5^) and for stimulus Set A (main effect of LEVEL OF DETAIL: F_1,16_ = 7.22, p = 0.016). In Figure 6 there seems to be a difference in prestimulus activity between real-world and non-sense object stimuli for stimulus Set B in the speeded response task. However, this difference was not statistically significant (t_16_<1.24, p>0.2).

### Late gamma band response

The lower panel of Figure 4 displays a late enhancement of total gamma band activity. The late gamma band response was also characterized by a clear peak in the time courses of total gamma activity (see Figure 6). This peak had a very broad spatial distribution and was more pronounced for schematic line drawings from stimulus Set A (main effect of LEVEL OF DETAIL: F_1,16_ = 4.80, p = 0.044, ANOVA details like early GBR). However, recognition related modulations of late total gamma activity were only observed for the more detailed images from stimulus Set B (LEVEL OF DETAIL×SEMANTIC CONTENT interaction: F_1,16_ = 5.11, p = 0.038, real-world vs. non-sense objects effect for stimulus Set A: t_16_ = −0.80, p = 0.438, real-world vs. non-sense objects effect for stimulus Set B: t_16_ = 2.65, p = 0.017).

## Discussion

In the current report, we investigated how different types of gamma band responses (GBRs) can be modulated depending on task requirements and stimulus' level of detail. Early evoked GBRs differentiated between real-world and non-sense objects, irrespective of the level of detail, only when participants had to perform speeded discriminations. Later induced GBRs differentiated between real-world and non-sense objects irrespective of response demands, only for sufficiently detailed stimulus material.

Previous studies related eGBRs to a fast processing mode based on temporal information [2], [20] that allows stimulus classifications within 100–150 ms after stimulus onset [22]. The current results confirm this hypothesis. Early differences between real-world and non-sense object stimuli become manifest in the temporal structure, i.e. the phase locking of the eGBRs. The results also indicate that this fast mode [20] seems to be used predominantly in those cases in which a speeded response was required. In situations that do not require a speeded response, additional information from a refinement system (presumably reflected by the iGBR) can further shape the response. The error rates in the current experiment are in line with this interpretation: Although a difference in the physiological response can be observed, the additional information contained in the more natural stimuli cannot completely be utilized in the speeded response task.

Reactions to the more detailed stimuli were generally faster than those to the line drawings, which supports previous findings [23]. This might indicate that participants benefit from the additional information contained in the natural stimuli. This suggests that although stimulus Set A was simpler from an information theoretic point of view, it might be less suited for the visual system and thus actually more difficult for the participants. This is also in line with the notion that the visual system is optimized for natural scenes [24], [25] which more closely resemble the stimuli in stimulus Set B. In the speeded response condition, participants had to first perceive the stimulus and use this information to immediately initiate a response. In contrast, in the delayed condition, participants could perceive the stimulus and prepare their response before the response screen appeared. If we assume that response execution takes approximately the same time in the speeded task and in the delayed task, the difference in reaction time gives a very coarse estimate of the time required to identify the stimulus and select a response in the speeded response task. This time, which is ∼180 ms, is clearly below the latency of the late gamma band response, yet still includes the early gamma band response. Thus, we infer that responses in the speeded response condition are based on a perceptual process that is reflected by the evoked GBR, whereas responses in the delayed response condition, might be initiated only after information from both early evoked and late total GBR has been integrated. Participants seemed to benefit from this additional information only for the more detailed stimuli.

The late gamma band amplitude modulation is comparable with respect to latency, frequency, and recognition modulation to what other authors have termed iGBR [12], [13], [14]. This seems to indicate that iGBRs only discriminate semantically meaningful objects from object-like but meaningless patterns if the stimuli provide a sufficient amount of detail. Interestingly, iGBRs have also been related to learning new stimuli [26], [27]. This is in line with the interpretation that iGBRs relate to a refinement system [2], the output of which could be used to modify future behavior [1]. Here, we propose that this refinement system is only activated if the stimuli are sufficiently detailed to support a further refinement. An example for stimuli that are not sufficiently detailed for further refinement seems to be given by the schematic line drawings.

In the speeded response condition, participants generally made more errors and differences between stimulus sets were less pronounced. Stimuli from both stimulus sets are similar on a very coarse scale (similar size, figure on background, etc.). Thus, we believe that in the speeded response condition, participants based their responses on coarse and global categorizations of the stimulus. This might indicate that the initiation of a button press can be based on such global categorizations even before all the details of a stimulus have been processed. A similar account comes from the reverse hierarchy theory [28], [29]. This theory states that incoming stimuli are rapidly relayed to higher visual areas. Conscious access to incoming stimuli then proceeds from global categorizations to successive levels of detail. The current findings link these two processing modes within the same system to evoked and induced GBRs [2], [5]. In an initial, fast but coarse, classification step, information is rapidly relayed to higher perceptual areas. This classification depends on the temporal fine structure of the spike wave triggered by the stimulus [2]. It has been suggested that such rapid processing could be mediated by the dorsal visual pathway [30], [31]. Different authors emphasized that initial, fast but coarse classifications should be based on feedforward processing [4], [29]. From a modeling study, Rodemann & Körner [32] inferred that the reliability of classifications by such a system strongly depends on the presence of evoked gamma oscillations. After this initial classification, feedback connections ensure that the information reverberates within the visual system [2], [4]. This leads to a refinement of the percept [1] and induced gamma band oscillations [2], [33]. These oscillations could, in turn, be used to adapt future behavior based on learning [26], [34]. The current findings demonstrate that these two modes can be modulated separately by fairly general experimental manipulations.

Previous reports that investigated recognition-related GBRs either found effects on evoked [10], [11] or on induced GBRs [12], [13], but not on both at the same time. The current results resolve this issue. The stimuli used by those authors that reported effects on eGBRs [10], [11] were probably too reduced to elicit significant effects on later iGBRs. Note that these stimuli are the same as those used in the present report in stimulus Set A. There seem to be different reasons why authors found recognition effects on iGBRs but not on eGBRs. Busch et al. [12] employed the same stimuli that we used in stimulus Set B in a delayed response task and reported effects on iGBRs, but not on eGBRs. It seems that the absence of a recognition effect on eGBRs in their data can be explained by the lack of time pressure in their experiment due to delayed response requirements. In the experiment by Gruber & Müller [13], no response delay was imposed. However, in that experiment, stimuli were reported to be relatively small (∼4.5×5.2°). Recent findings suggest that eGBRs are highly dependent on stimulus parameters, in particular size [6], [7]. Furthermore, the size of a stimulus not only seems to be a prerequisite for reliably measuring eGBRs but also for detecting top-down effects on eGBRs [8]. Thus, it might be expected that the stimuli employed by Gruber & Müller [13] were not sufficiently large to evoke a detectable eGBR effect.

In conclusion, we were able to demonstrate that two different visual processing modes can be discriminated. One of these modes mediates rapid, but less accurate, categorization processes and seems to be based mainly on temporal relations to the stimulus as quantified by evoked gamma band responses. The other mode is slower, but more accurate, and seems to be based on temporal relations between neural groups as quantified by induced gamma band responses.

## Materials and Methods

### Participants

Seventeen healthy volunteers (mean age: 23.76+/−2.34 years, range: 20 to 28 years, 5 m, 12 f) participated in the current study. Participants did not report any current or past psychiatric or neurological disorders and received money or course credits for their participation. Before the experiment, participants were informed about the procedure and the aim of the study. They all gave written consent for their participation. The experimental procedure was in accordance with the Declaration of Helsinki as well as with the guidelines of the local ethics committee of the Otto-von-Guericke University of Magdeburg.

### Stimuli and experimental procedure

Participants were required to categorize real-world objects and object-like but meaningless patterns (non-sense objects) as either meaningful or meaningless. In the speeded response condition they were required to select the correct button as fast as possible, whereas in the delayed response task, no time pressure was imposed (see below). These two tasks were performed on two different sets of stimuli. One set of stimuli contained black and white line drawings of real-world and non-sense objects (stimulus Set A, see left column of Figure 1). Another set of stimuli contained more detailed, colored pictures of real-world and non-sense objects (stimulus Set B, see right column of Figure 1).

Stimuli in Set A were all high contrast schematic black on white drawings. Non-sense objects were constructed by rearranging the lines from the real-world objects. This way, the number of black and white pixels and the number of black and white edges was approximately the same for real-world and non-sense object stimuli. A detailed description of the stimuli in Set A can be found elsewhere [10]. Examples for stimuli from stimulus Set A are shown on the left side of Figure 1. Stimuli in Set B were derived from images of natural objects. To obtain a set of non-sense object stimuli, these images were distorted. From this set of original and distorted images, stimulus Set B was derived by averaging the amplitude spectra of spatial frequencies and differentiating the stimuli only by means of their phase spectra. A detailed description of the stimuli in Set B can be found elsewhere [12]. Examples for stimuli from stimulus Set B are shown in the right column of Figure 1.

We quantified the level of detail of the stimuli by means of the Shannon entropy
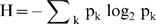
where p_k_ denotes the relative frequency of the pixel luminance k in the given stimulus set. The index k ran over red, green and blue color bands independently. The average entropy for stimuli from stimulus Set A was 2.67 bit/pixel, the average entropy for stimuli from stimulus Set B was 8.64 bit/pixel. Thus, at least in principle, more information could be extracted from stimuli in stimulus Set B than from stimuli in stimulus Set A.

Each set of stimuli was presented in two blocks of 200 stimuli each (100 real-world objects and 100 non-sense objects). Participants were instructed to press a button with one hand to indicate that the current stimulus represented a meaningful real-world object and to press another button with the other hand to indicate that the current stimulus represented a meaningless non-sense object. In one of the two blocks, participants were to press the button as quickly as possible (speeded response). In the other block, they were to press the button only after a response screen had been presented one second after the onset of the stimulus (delayed response). The response screen had the same medium gray color as the background during the whole experiment. It consisted of a written instruction to respond. This instruction was presented in black letters. In total, each participant responded to a total of four blocks: one block with stimuli from Set A and speeded response requirements, one block with stimuli from Set A and delayed response requirements, one block with stimuli from Set B and speeded response requirements, and one block with stimuli from Set B and delayed response requirements. Each block was preceded by a practice block of 16 trials that was not analyzed. During the practice block, participants were able to become familiar with the stimuli and the task demands of the new block. Block sequence and response buttons were counterbalanced across participants.

Stimuli were presented on a 24” TFT-display at a distance of 122 cm. The latency of the display was measured using a photodiode. The monitor responded with a delay of 48 ms (first deviation from noise, standard deviation less than the EEG sampling interval of 2 ms). All trigger information that was used for the subsequent EEG analysis was shifted to compensate for this delay. The stimuli subtended a region of ∼8 to 10 degrees visual angle which has been shown to be suitable to evoke GBRs [6], [7]. The stimuli were presented in random order with interstimulus intervals drawn from a uniform distribution between 1000 and 2000 ms. Stimulus duration was 1000 ms in the speeded response task and 500 ms in the delayed response task. In the delayed response blocks, there was a 500 ms delay after each stimulus before the response screen was presented. This way, block duration was kept approximately constant in order to avoid fatigue effects. Participants were instructed to fixate a small black cross that was presented at the center of the presentation screen.

### Data acquisition

Participants performed the experiment in an electrically shielded, sound-attenuated, and dimly lit cabin (IAC, Niederkrüchten, Germany). The stimulation monitor was placed outside the cabin behind an electrically shielded window. All devices inside the cabin were battery-operated to avoid line frequency interference (50 Hz in Germany). The electroencephalogram (EEG) was measured from 31 scalp locations according to an extended 10–20 system and amplified using a BrainAmp amplifier (Brain Products, Munich, Germany). An electrode placed on the nose served as reference. In order to detect artifacts due to eye movements, an electrode placed below the orbital rim was used to record the electrooculogram (EOG). Activity was recorded using sintered Ag/AgCl electrodes mounted in an elastic cap (Easycap, Falk Minow, Munich, Germany). Electrode impedances were kept below 5 kΩ . EEG-Data were acquired with a band-pass filter of 0.016–250 Hz and a sampling rate of 500 Hz. A fiber optic cable transferred the digitized EEG to a computer outside the recording cabin. A digital high pass filter with a cutoff frequency of 0.5 Hz was applied offline in order to reduce slow shifts in the baseline. If participants moved their eyes away from the fixation cross, it was detected by measurements of EOG activity and the trial was discarded. For this purpose, an automatic artifact detection was computed, which excluded trials from further analysis if the standard deviation within a moving 200 ms window exceeded 40 µV in any channel. The automatic artifact rejection was supplemented by visual inspection of every trial to ensure that only trials without artifacts were included in the subsequent analysis.

### Data analysis

The percentage of correct responses was ascertained for all trials. In addition, mean reaction times were determined for speeded response trials with respect to stimulus onset. Mean reaction times were determined for delayed response trials with respect to the onset of the response display (1000 ms after stimulus onset).

Event related potentials (ERPs) were computed as averages of all artifact-free trials of a given condition. These curves were aligned by subtracting baseline activity from the last 200 ms preceding stimulus onset. Grand average time courses were computed by averaging ERP waveforms from all participants.

Gamma band responses were characterized by three parameters derived from the EEG by means of the wavelet transform (Morlet wavelet, time frequency localization at 40 Hz: 2σ_t_∼50 ms, 2σ_f_∼13 Hz). The wavelet transform was evaluated for center frequencies ranging in steps of one Hz from 1 to 90 Hz. If the grid, on which a wavelet had been evaluated, extended over the borders of a trial, the signal was padded with zeros. The convolutions required to evaluate the wavelet transform were performed using custom made software written in C. Amplitudes were scaled to conserve the amplitudes of sine wave test signals, rather than total signal energy. Thus, the wavelet transform at a single frequency corresponded to a filtered analytical version of the original signal. The three parameters extracted from the wavelet transform were: (i) The evoked activity, which is the amplitude of the wavelet transform of the ERP; (ii) the total activity, which is the averaged absolute amplitude of the single trial wavelet transforms; and (iii) the degree of phase locking (PL) to the stimulus quantified as the mean resultant length [35] of the single trial phases. Thus, the phase locking values range from 0 to 1. A value of 1 indicates perfect phase alignment across single trials; a value of 0 implies that the trials are not phase locked in such a way that they cancel out in the evoked potential. A more detailed description of these parameters can be found in Herrmann & Mecklinger [36] and Herrmann et al. [37].

It has been demonstrated that the exact frequency of the GBR varies in a very consistent manner across participants [7]. To account for frequency variations across participants, the time frequency planes from each participant were averaged across all conditions. The response frequency was defined as the frequency that displayed the strongest deviation from a baseline (200 to 100 ms before stimulus onset). Two different response frequencies were ascertained: the frequency of the eGBR, as local maximum of evoked activity in the time range between 60 and 140 ms after stimulus onset and the frequency of the iGBR as local maximum of total activity in the time range between 200 and 400 ms after stimulus onset. In the former time range, first feedback effects can be expected [38], while in the latter time range, high-level object related effects have been described [39]. In both cases, response frequencies were manually selected from the frequency range between 30 and 90 Hz. A response frequency could be ascertained unambiguously in most cases. If for a particular participant no response frequency could be ascertained, a frequency of 40 Hz was selected. Response frequencies of the eGBR were between 32 and 66 Hz (mean 42 Hz) and response frequencies for iGBR were between 35 and 69 Hz (mean 50 Hz). Response frequencies generally did not differ significantly between conditions. An exception was a significant difference in response frequency of the eGBR between the two stimulus sets (F_1,14_ = 4.65, p = 0.049, Set A: 45.4+/−5.4 Hz, Set B: 41.0+/−4.8 Hz). In this article, we report results for one frequency for early and another frequency for late responses only. Time courses of evoked and total activity as well as PL were extracted at these two frequencies.

To avoid loss of statistical power, electrodes were pooled into regions of interest (ROI). Responses were evaluated from a posterior ROI (electrodes O1, O2, P7, P3, Pz, P4 and P8) and from a central ROI (electrodes CP1, CP2, C3, Cz, C4, FC1, FC2). These ROIs were chosen from those electrodes that displayed a strong signal change after stimulation. Repeated measurements analyses of variance were used to judge the statistical significance of the factors TIME PRESSURE (speeded vs. delayed response), SEMANTIC CONTENT (real-world vs. non-sense object), LEVEL OF DETAIL (schematic line drawings from Set A vs. detailed images from Set B), and ROI. Separate analyses of variance were performed on the percentage of correct responses (without the factor ROI), on mean ERP amplitude between 250 and 400 ms, and on early (mean amplitude between 60 and 140 ms) and late (mean amplitude between 200 and 400 ms) GBR. Phase locking factors were transformed to Fisher's z values via z = 0.5 log((1+PLF)/(1-PLF)) before statistical analyses. As all factors in the analyses of variance had two levels only, corrections for inhomogeneities of covariance were not necessary [40].
